# Methyl 2-[(*E*)-3-hydr­oxy-4-methoxy­benzyl­idene]hydrazinecarboxyl­ate

**DOI:** 10.1107/S1600536809018996

**Published:** 2009-05-23

**Authors:** Lu-Ping Lv, Tie-Ming Yu, Wen-Bo Yu, Wei-Wei Li, Xian-Chao Hu

**Affiliations:** a Department of Chemical Engineering, Hangzhou Vocational and Technical College, Hangzhou 310018, People’s Republic of China; b Research Center of Analysis and Measurement, Zhejiang University of Technology, Hangzhou 310014, People’s Republic of China

## Abstract

The title compound, C_10_H_12_N_2_O_4_, adopts a *trans* configuration with respect to the C=N bond. The hydrazinecarboxyl­ate group is twisted from the benzene ring by 6.62 (5)° and an intramolecular O—H⋯O hydrogen bond occurs. In the crystal structure, mol­ecules are linked into a two-dimensional network parallel to (100) by O—H⋯O, N—H⋯O and C—H⋯O hydrogen bonds. In addition,  weak C—H⋯π inter­actions are observed.

## Related literature

For properties of benzaldehyde­hydrazone derivatives, see: Parashar *et al.* (1988 ▶); Hadjoudis *et al.* (1987 ▶); Borg *et al.* (1999 ▶). For Schiff base metal complexes, see: Kahwa *et al.* (1986 ▶); Santos *et al.* (2001 ▶). For a related structure, see: Shang *et al.* (2007 ▶).
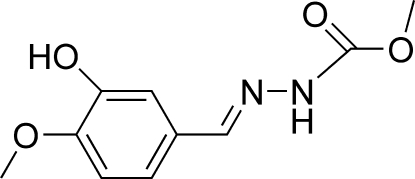


## Experimental

### 

#### Crystal data

C_10_H_12_N_2_O_4_
*M* *_r_* = 224.22
Monoclinic, 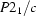

*a* = 7.7223 (12) Å
*b* = 9.2106 (14) Å
*c* = 15.092 (2) Å
β = 100.944 (6)°
*V* = 1054.0 (3) Å^3^
*Z* = 4
Mo *K*α radiation
μ = 0.11 mm^−1^
*T* = 223 K
0.18 × 0.16 × 0.15 mm

#### Data collection

Bruker SMART CCD area-detector diffractometer
Absorption correction: multi-scan (*SADABS*; Bruker, 2002 ▶) *T*_min_ = 0.978, *T*_max_ = 0.982
5767 measured reflections
1944 independent reflections
1657 reflections with *I* > 2σ(*I*)
*R*_int_ = 0.024

#### Refinement

*R*[*F* ^2^ > 2σ(*F* ^2^)] = 0.034
*wR*(*F* ^2^) = 0.098
*S* = 1.05
1944 reflections
149 parameters
H-atom parameters constrained
Δρ_max_ = 0.19 e Å^−3^
Δρ_min_ = −0.18 e Å^−3^

### 

Data collection: *SMART* (Bruker, 2002 ▶); cell refinement: *SAINT* (Bruker, 2002 ▶); data reduction: *SAINT*; program(s) used to solve structure: *SHELXS97* (Sheldrick, 2008 ▶); program(s) used to refine structure: *SHELXL97* (Sheldrick, 2008 ▶); molecular graphics: *SHELXTL* (Sheldrick, 2008 ▶); software used to prepare material for publication: *SHELXTL*.

## Supplementary Material

Crystal structure: contains datablocks I, global. DOI: 10.1107/S1600536809018996/ci2804sup1.cif

Structure factors: contains datablocks I. DOI: 10.1107/S1600536809018996/ci2804Isup2.hkl

Additional supplementary materials:  crystallographic information; 3D view; checkCIF report

## Figures and Tables

**Table 1:** Hydrogen-bond geometry (Å, °)

*D*—H⋯*A*	*D*—H	H⋯*A*	*D*⋯*A*	*D*—H⋯*A*
O2—H2⋯O1	0.82	2.28	2.6871 (13)	112
O2—H2⋯O3^i^	0.82	2.20	2.9303 (13)	148
N2—H2*A*⋯O3^ii^	0.86	2.44	3.1951 (15)	147
C8—H8⋯O3^ii^	0.93	2.51	3.3185 (16)	146
C10—H10*A*⋯*Cg*1^iii^	0.96	2.87	3.6878 (18)	143

Symmetry codes: (i) 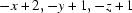; (ii) 
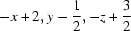
; (iii) 
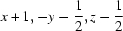
. *Cg*1 is the centroid of the C2–C7 ring.

**Fig. 1.**
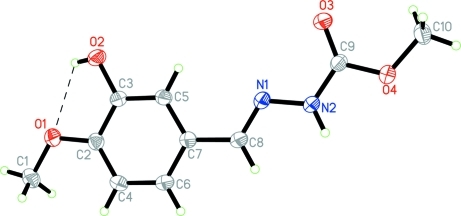
The molecular structure of the title compound. Displacement ellipsoids are drawn at the 30% probability level. The dashed line indicates a hydrogen bond.

**Fig. 2.**
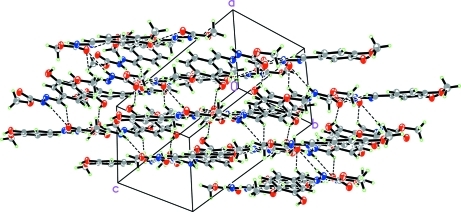
Crystal packing of the title compound. Hydrogen bonds are shown as dashed lines.

## supplementary crystallographic information

### Comment

Benzaldehydehydrazone derivatives have attracted much attention due to their pharmacological activity (Parashar *et al.*, 1988) and their photochromic properties (Hadjoudis *et al.*, 1987). They are important intermediates of 1,3,4-oxadiazoles, which have been reported to be versatile compounds with many interesting properties (Borg *et al.*, 1999). Metal complexes based on Schiff bases have received considerable attention because they can be utilized as model compounds of active centres in various proteins and enzymes (Kahwa *et al.*, 1986; Santos *et al.*, 2001). We report here the crystal structure of the title compound (Fig. 1).

The title molecule adopts a *trans* configuration with respect to the C═N bond. The hydrazinecarboxylate group is twisted from the benzene ring by 6.62 (5)°. The bond lengths and angles are comparable to those observed for methyl*N*'-[(*E*)-4-methoxybenzylidene]hydrazinecarboxylate (Shang *et al.*, 2007). An intramolecular O—H···O interaction is observed.

In the crystal structure, the molecules are linked into a two-dimensional network parallel to the (100) by O—H···O, N—H···O and C—H···O hydrogen bonds (Table 1 and Fig.2). In addition, a C—H···π interaction is observed.

### Experimental

3-Hydroxy-4-methoxy-benzaldehyde (1.52 g, 0.01 mol) and methyl hydrazinecarboxylate (0.90 g, 0.01 mol) were dissolved in stirred methanol (25 ml) and left for 4 h at room temperature. The resulting solid was filtered off and recrystallized from ethanol to give the title compound in 75% yield. Single crystals suitable for X-ray analysis were obtained by slow evaporation of an ethanol solution at room temperature (m.p. 398–401 K).

### Refinement

H atoms were positioned geometrically (N-H = 0.86 Å and C-H = 0.93 or 0.96 Å) and refined using a riding model, with *U*_iso_(H) = 1.2*U*_eq_(C,N) and 1.5*U*_eq_(C_methyl_).

### Crystal data

**Table:**

C_10_H_12_N_2_O_4_	*F*(000) = 472
*M**_r_* = 224.22	*D*_x_ = 1.413 Mg m^−^^3^
Monoclinic, *P*2_1_/*c*	Mo *K*α radiation, λ = 0.71073 Å
Hall symbol: -P 2ybc	Cell parameters from 1944 reflections
*a* = 7.7223 (12) Å	θ = 2.6–25.5°
*b* = 9.2106 (14) Å	µ = 0.11 mm^−^^1^
*c* = 15.092 (2) Å	*T* = 223 K
β = 100.944 (6)°	Block, colourless
*V* = 1054.0 (3) Å^3^	0.18 × 0.16 × 0.15 mm
*Z* = 4	

### Data collection

**Table:**

Bruker SMART CCD area-detector diffractometer	1944 independent reflections
Radiation source: fine-focus sealed tube	1657 reflections with *I* > 2σ(*I*)
graphite	*R*_int_ = 0.024
φ and ω scans	θ_max_ = 25.5°, θ_min_ = 2.6°
Absorption correction: multi-scan (*SADABS*; Bruker, 2002)	*h* = −9→8
*T*_min_ = 0.978, *T*_max_ = 0.982	*k* = −11→11
5767 measured reflections	*l* = −18→18

### Refinement

**Table:**

Refinement on *F*^2^	Secondary atom site location: difference Fourier map
Least-squares matrix: full	Hydrogen site location: inferred from neighbouring sites
*R*[*F*^2^ > 2σ(*F*^2^)] = 0.034	H-atom parameters constrained
*wR*(*F*^2^) = 0.098	*w* = 1/[σ^2^(*F*_o_^2^) + (0.0545*P*)^2^ + 0.1597*P*] where *P* = (*F*_o_^2^ + 2*F*_c_^2^)/3
*S* = 1.05	(Δ/σ)_max_ = 0.001
1944 reflections	Δρ_max_ = 0.19 e Å^−^^3^
149 parameters	Δρ_min_ = −0.18 e Å^−^^3^
0 restraints	Extinction correction: *SHELXL97* (Sheldrick, 2008), Fc^*^=kFc[1+0.001xFc^2^λ^3^/sin(2θ)]^-1/4^
Primary atom site location: structure-invariant direct methods	Extinction coefficient: 0.035 (4)

### Special details

**Table:**

Geometry. All e.s.d.'s (except the e.s.d. in the dihedral angle between two l.s. planes) are estimated using the full covariance matrix. The cell e.s.d.'s are taken into account individually in the estimation of e.s.d.'s in distances, angles and torsion angles; correlations between e.s.d.'s in cell parameters are only used when they are defined by crystal symmetry. An approximate (isotropic) treatment of cell e.s.d.'s is used for estimating e.s.d.'s involving l.s. planes.
Refinement. Refinement of *F*^2^ against ALL reflections. The weighted *R*-factor *wR* and goodness of fit *S* are based on *F*^2^, conventional *R*-factors *R* are based on *F*, with *F* set to zero for negative *F*^2^. The threshold expression of *F*^2^ > σ(*F*^2^) is used only for calculating *R*-factors(gt) *etc*. and is not relevant to the choice of reflections for refinement. *R*-factors based on *F*^2^ are statistically about twice as large as those based on *F*, and *R*- factors based on ALL data will be even larger.

### Fractional atomic coordinates and isotropic or equivalent isotropic displacement parameters (Å^2^)

**Table:**

	*x*	*y*	*z*	*U*_iso_*/*U*_eq_	
C9	1.14900 (17)	0.19752 (13)	0.79259 (8)	0.0395 (3)	
C8	0.89788 (18)	0.11117 (13)	0.58309 (9)	0.0435 (3)	
H8	0.8814	0.0133	0.5941	0.052*	
C3	0.78128 (16)	0.37223 (13)	0.39017 (8)	0.0386 (3)	
C5	0.84592 (17)	0.31881 (13)	0.47483 (8)	0.0392 (3)	
H5	0.9053	0.3802	0.5193	0.047*	
C7	0.82305 (17)	0.17208 (13)	0.49464 (8)	0.0399 (3)	
C2	0.68958 (16)	0.28075 (14)	0.32243 (8)	0.0401 (3)	
C6	0.73162 (19)	0.08300 (14)	0.42746 (9)	0.0475 (3)	
H6	0.7149	−0.0143	0.4401	0.057*	
C4	0.66479 (18)	0.13636 (14)	0.34190 (9)	0.0466 (3)	
H4	0.6035	0.0753	0.2978	0.056*	
C10	1.3093 (2)	0.17575 (17)	0.94154 (9)	0.0564 (4)	
H10A	1.3936	0.2432	0.9261	0.085*	
H10B	1.3697	0.1025	0.9810	0.085*	
H10C	1.2281	0.2263	0.9714	0.085*	
C1	0.5523 (2)	0.25850 (18)	0.16765 (10)	0.0575 (4)	
H1A	0.6295	0.1791	0.1614	0.086*	
H1B	0.5317	0.3150	0.1132	0.086*	
H1C	0.4422	0.2214	0.1787	0.086*	
O1	0.63158 (13)	0.34761 (10)	0.24138 (6)	0.0500 (3)	
O4	1.21426 (13)	0.10865 (10)	0.86081 (6)	0.0513 (3)	
O3	1.17310 (14)	0.32803 (9)	0.79258 (6)	0.0529 (3)	
O2	0.80571 (14)	0.51620 (9)	0.37432 (6)	0.0520 (3)	
H2	0.7894	0.5307	0.3197	0.078*	
N1	0.98497 (14)	0.18969 (11)	0.64511 (7)	0.0420 (3)	
N2	1.05406 (15)	0.12010 (11)	0.72457 (7)	0.0442 (3)	
H2A	1.0369	0.0287	0.7307	0.053*	

### Atomic displacement parameters (Å^2^)

**Table:**

	*U*^11^	*U*^22^	*U*^33^	*U*^12^	*U*^13^	*U*^23^
C9	0.0475 (7)	0.0337 (6)	0.0380 (7)	0.0026 (5)	0.0096 (5)	0.0032 (5)
C8	0.0531 (8)	0.0325 (6)	0.0440 (7)	−0.0037 (5)	0.0067 (6)	0.0023 (5)
C3	0.0427 (7)	0.0331 (6)	0.0406 (7)	0.0000 (5)	0.0091 (5)	−0.0006 (5)
C5	0.0441 (7)	0.0354 (6)	0.0372 (6)	−0.0039 (5)	0.0057 (5)	−0.0032 (5)
C7	0.0425 (7)	0.0369 (6)	0.0397 (7)	−0.0015 (5)	0.0067 (5)	0.0002 (5)
C2	0.0412 (7)	0.0415 (7)	0.0369 (7)	0.0025 (5)	0.0060 (5)	−0.0007 (5)
C6	0.0571 (8)	0.0335 (6)	0.0497 (8)	−0.0070 (6)	0.0045 (6)	−0.0007 (5)
C4	0.0518 (8)	0.0417 (7)	0.0430 (7)	−0.0061 (6)	0.0003 (6)	−0.0079 (5)
C10	0.0626 (9)	0.0631 (9)	0.0391 (8)	−0.0074 (7)	−0.0015 (6)	0.0067 (6)
C1	0.0618 (9)	0.0646 (9)	0.0411 (8)	−0.0054 (7)	−0.0030 (6)	−0.0032 (7)
O1	0.0606 (6)	0.0474 (5)	0.0377 (5)	0.0009 (4)	−0.0018 (4)	0.0008 (4)
O4	0.0664 (6)	0.0407 (5)	0.0416 (5)	−0.0009 (4)	−0.0027 (4)	0.0066 (4)
O3	0.0776 (7)	0.0341 (5)	0.0440 (5)	−0.0049 (4)	0.0038 (5)	0.0007 (4)
O2	0.0772 (7)	0.0348 (5)	0.0415 (5)	−0.0048 (4)	0.0053 (5)	0.0044 (4)
N1	0.0517 (6)	0.0348 (5)	0.0380 (6)	0.0009 (4)	0.0049 (5)	0.0046 (4)
N2	0.0602 (7)	0.0300 (5)	0.0393 (6)	−0.0015 (5)	0.0015 (5)	0.0040 (4)

### Geometric parameters (Å, °)

**Table:**

C9—O3	1.2164 (15)	C6—C4	1.3864 (19)
C9—O4	1.3369 (15)	C6—H6	0.93
C9—N2	1.3469 (16)	C4—H4	0.93
C8—N1	1.2698 (16)	C10—O4	1.4371 (16)
C8—C7	1.4621 (17)	C10—H10A	0.96
C8—H8	0.93	C10—H10B	0.96
C3—O2	1.3669 (14)	C10—H10C	0.96
C3—C5	1.3717 (17)	C1—O1	1.4241 (16)
C3—C2	1.4078 (17)	C1—H1A	0.96
C5—C7	1.4025 (17)	C1—H1B	0.96
C5—H5	0.93	C1—H1C	0.96
C7—C6	1.3885 (18)	O2—H2	0.82
C2—O1	1.3668 (15)	N1—N2	1.3750 (14)
C2—C4	1.3831 (19)	N2—H2A	0.86
			
O3—C9—O4	124.74 (12)	C2—C4—H4	120.1
O3—C9—N2	125.75 (11)	C6—C4—H4	120.1
O4—C9—N2	109.51 (10)	O4—C10—H10A	109.5
N1—C8—C7	121.11 (11)	O4—C10—H10B	109.5
N1—C8—H8	119.4	H10A—C10—H10B	109.5
C7—C8—H8	119.4	O4—C10—H10C	109.5
O2—C3—C5	118.24 (11)	H10A—C10—H10C	109.5
O2—C3—C2	121.38 (11)	H10B—C10—H10C	109.5
C5—C3—C2	120.37 (11)	O1—C1—H1A	109.5
C3—C5—C7	120.40 (11)	O1—C1—H1B	109.5
C3—C5—H5	119.8	H1A—C1—H1B	109.5
C7—C5—H5	119.8	O1—C1—H1C	109.5
C6—C7—C5	118.74 (12)	H1A—C1—H1C	109.5
C6—C7—C8	119.87 (11)	H1B—C1—H1C	109.5
C5—C7—C8	121.36 (11)	C2—O1—C1	117.31 (11)
O1—C2—C4	126.04 (11)	C9—O4—C10	116.53 (10)
O1—C2—C3	114.48 (11)	C3—O2—H2	109.5
C4—C2—C3	119.47 (11)	C8—N1—N2	116.16 (10)
C4—C6—C7	121.22 (12)	C9—N2—N1	118.88 (10)
C4—C6—H6	119.4	C9—N2—H2A	120.6
C7—C6—H6	119.4	N1—N2—H2A	120.6
C2—C4—C6	119.79 (12)		

### Hydrogen-bond geometry (Å, °)

**Table:**

*D*—H···*A*	*D*—H	H···*A*	*D*···*A*	*D*—H···*A*
O2—H2···O1	0.82	2.28	2.6871 (13)	112
O2—H2···O3^i^	0.82	2.20	2.9303 (13)	148
N2—H2A···O3^ii^	0.86	2.44	3.1951 (15)	147
C8—H8···O3^ii^	0.93	2.51	3.3185 (16)	146
C10—H10A···Cg1^iii^	0.96	2.87	3.6878 (18)	143

Symmetry codes: (i) −*x*+2, −*y*+1, −*z*+1; (ii) −*x*+2, *y*−1/2, −*z*+3/2; (iii) *x*+1, −*y*−1/2, *z*−1/2.
